# Dental Caries and Associated Factors in 3–5-Year-Old Children in Guizhou Province, China: An Epidemiological Survey (2015–2016)

**DOI:** 10.3389/fpubh.2021.747371

**Published:** 2021-09-30

**Authors:** Min Guan, Ola A. Nada, Juan-juan Wu, Jiang-ling Sun, Na Li, Li-ming Chen, Tai-ming Dai

**Affiliations:** ^1^Department of Conservative Dentistry and Endodontics, Guiyang Stomatological Hospital, Guiyang, China; ^2^Oral Biology Department, Faculty of Dentistry, Alexandria University, Alexandria, Egypt; ^3^Department of Medicine, Faculty of Medicine, Guizhou University, Guiyang, China

**Keywords:** early childhood caries, associated factors, epidemiological survey, pre-school children, design efficiency deff

## Abstract

**Objective:** This study aimed to explore the factors influencing dental caries among 3–5-year-old children in Guizhou Province and the interrelationship between these factors using structural equation modeling, while providing theoretical references to improve the prevention and control strategy.

**Materials and Methods:** A total of 1,291 children aged 3–5 years in Guizhou Province were selected by a multistage stratified and whole group random sampling to examine the caries prevalence in whole-mouth deciduous teeth crowns, and parents were surveyed with questionnaires to analyze the caries-related factors. IBM SPSS Statistics v 23.0 software (IBM, Armonk, NY, USA) was used for statistical analysis.

**Results:** The caries prevalence of children aged 3–5 years in Guizhou Province was 63.1%, the mean decayed-missing-filled teeth was 3.32, the caries filling rate was 0.5%, and there was no statistically significant difference between urban and rural areas and among genders in each age group; results of logistic regression analysis showed that the caries risk increased with the following factors: age, brushing frequency <2 times per day when parents did not take their children to the dentist, and with parents poor evaluation of the oral condition of their children. The higher the education of the parent, the lower the risk of children suffering from caries in deciduous teeth.

**Conclusions:** With an overall poor situation about oral hygiene habits, oral healthcare attitude of the parents, and behavior transformation, the prevalence of dental caries in the deciduous teeth of children aged 3–5 years in Guizhou Province is high, and their caries status was severe, with more than 99% of the caries cases that were untreated. Therefore, prevention and treatment measures of caries in preschool children need strengthening through the improvement of public awareness and the enhancement of the management of oral health habits of their children.

## Introduction

Dental caries is one of the most common chronic diseases among children in the world, particularly in developing countries during the past decades (1, 2). Early childhood caries (ECC) is defined as the presence of decayed, missing, and filled tooth surfaces in any deciduous dentition occurring in a child younger than 71 months, which is an oral disease that is influenced by several factors, such as socioeconomic factors, dietary factors, oral health behaviors, and biological factors (3–7). Although the rapid economic development in China in the last decade has changed the oral health condition of the residents, survey results in some regions still revealed that the caries prevalence in preschool children is high (8–10). Guizhou Province is located in an economically underdeveloped area with a concentration of ethnic minorities in western China. Through the implementation of national economic stimulation policies such as “Western Development,” “One Belt and One Road,” and “Poverty Alleviation,” Guizhou Province has been ranked among the top in the country in terms of economic growth rates for several years where the economic situation and living conditions of the residents have improved. In the context of changing socioeconomic factors, the changes in the prevalence and influencing factors of ECC in Guizhou Province citizens deserve attention. In this study, a sample of children aged 3–5 years in 12 kindergartens belonging to two districts (Huichuan District, Zunyi City, and Xixiu District, Anshun City) and two counties (Zunyi County, Zunyi City, and Hezhang County, Bijie Region) in the Guizhou Province was surveyed for their oral health status. Moreover, influencing factors associated with ECC were analyzed to provide a reference for oral health policies referencing the oral prevention measures and treatment strategies in Guizhou Province.

## Materials and Methods

### Object of This Investigation

This study was conducted in Guizhou Province from October 2015 to May 2016 as a part of the Fourth National Oral Health Epidemiological Survey in China. This study was approved by the Dental Ethics Committee of the Chinese Society of Stomatology on July 9, 2014 (approval number: 2014-003). The survey population included children aged 3-, 4-, and 5-year-old [age was calculated according to the month of the survey], and according to the sample size formula, design efficiency (deff) = 4.5, with μ the confidence level and α set to 0.05. The estimated rate *p* was set to 66.0% according to the caries prevalence of 5-year-old children with deciduous teeth (according to the Third National Oral Health Epidemiological Survey), δ error margin was 10%, and the non-response rate was 20%.

The multistage stratified, cluster random sampling method was used, and two districts and two counties in Guizhou Province were randomly selected according to the population proportion using probability proportionate to size sampling method; then, three kindergartens were randomly selected from each district (county), totaling 12 kindergartens. Among the selected kindergartens, 1,291 subjects were selected by random sampling method from eligible children aged 3, 4, and 5 years using the cluster sampling method. Kindergartens with insufficient sample sizes were filled from neighboring kindergartens.

### Survey Content and Methods

#### Oral Examination

The examination was conducted by the dentist, who administered them inside the kindergartens that provided a quiet room. Participants took the sitting position during examination under the artificial light of the dental chair. Written informed consent was obtained from the legal guardians of participants before the survey. Referring to the 5th edition of Basic Methods of Oral Health Survey of WHO and the Fourth National Oral Health Epidemiological Survey Program, the caries status of the crowns of 20 deciduous teeth in the oral cavity of children aged 3–5 years was examined by visual examination combined with probing under artificial light with a dental mirror and a WHO periodontal probe. Caries prevalence was recorded as decayed-missing-filled teeth (dmft) > 0. The DMFT/dmft index was recorded according to WHO guidelines.

#### Questionnaire Survey

The questionnaires were obtained from the Fourth National Oral Health Epidemiological Survey. It was completed by one-on-one on-site questioning the parents of children by questionnaire investigators at the agreed time and place. The questionnaires included general information (name, age, gender, residence, and survey dates), attitudes and behaviors, and other related situations. The oral health attitude survey had six questions, of which 2 points were given for favorable attitude, 1 point for neutral, and 0 points for indifferent attitude.

### Quality Control of Inspection and Questionnaire Personnel

The oral examiners were all dentists with more than 3 years of clinical work experience, who received theoretical and clinical operation training before the survey, with a standard consistency test Kappa value of caries examination at 0.82–0.92. During the on-site examination, the respondents were randomly selected according to the 5% reexamination rate and were reexamined by another examiner to calculate the Kappa value ≥ 0.8. The questionnaire personnel also received training and were subjected to standard consistency tests after the training, and the questionnaire answers all had a compliance rate of 95% or more.

### Statistical Analysis

IBM SPSS Statistics v 23.0 software (IBM, Armonk, NY, USA) was used for statistical analysis. The filling rate and the significant caries index (SiC) were calculated. Quantitative variables were compared using *t*-test or ANOVA, while qualitative variables, such as factors related to caries in deciduous *teeth*, were compared through chi-squared test and multivariable logistic regression (LR) analysis. The level of statistical testing was set at 0.05.

### Strengthening the Reporting of Observational Studies in Epidemiology Guidelines

This study adheres to the STROBE guidelines.

## Results

### Oral Examination

The caries prevalence, dmft, SiC, and caries filling ratio of 1,291 children aged 3–5 years in Guizhou Province are shown in Table 1, and the differences between the urban and rural areas and among genders were not found to be statistically significant (*p* > 0.05).

**Table 1:** Dental caries status of 3- to 5-year-old children in Guizhou Province.

**Item**	**Number**	**The prevalence (%) of caries**	***P*** **-value**	**The mean dmft**	***P*** **-value**	**Significant caries index (SiC)**	***P*** **-value**	**Caries filling rate (%)**	***P*** **-value**
**Residence**									
Urban	645	65.1	0.125	3.54	0.049	7.98	0.404	0.5	0.909
Rural	646	61.0		3.09		8.28		0.6	
**Gender**									
Male	639	62.1	0.496	3.34	0.849	8.32	0.238	0.2	0.092
Female	652	64.0		3.30		7.90		0.9	
**Age**									
3 (years)	410	51.2	0.000	2.44	0.000	6.51	0.152	0.2	0.556
4 (years)	442	66.5		3.62		8.76		0.5	
5 (years)	439	70.6		3.83		8.68		0.9	
Total	1,291	63.1		3.32		8.11		0.5	

**Figure 1:**
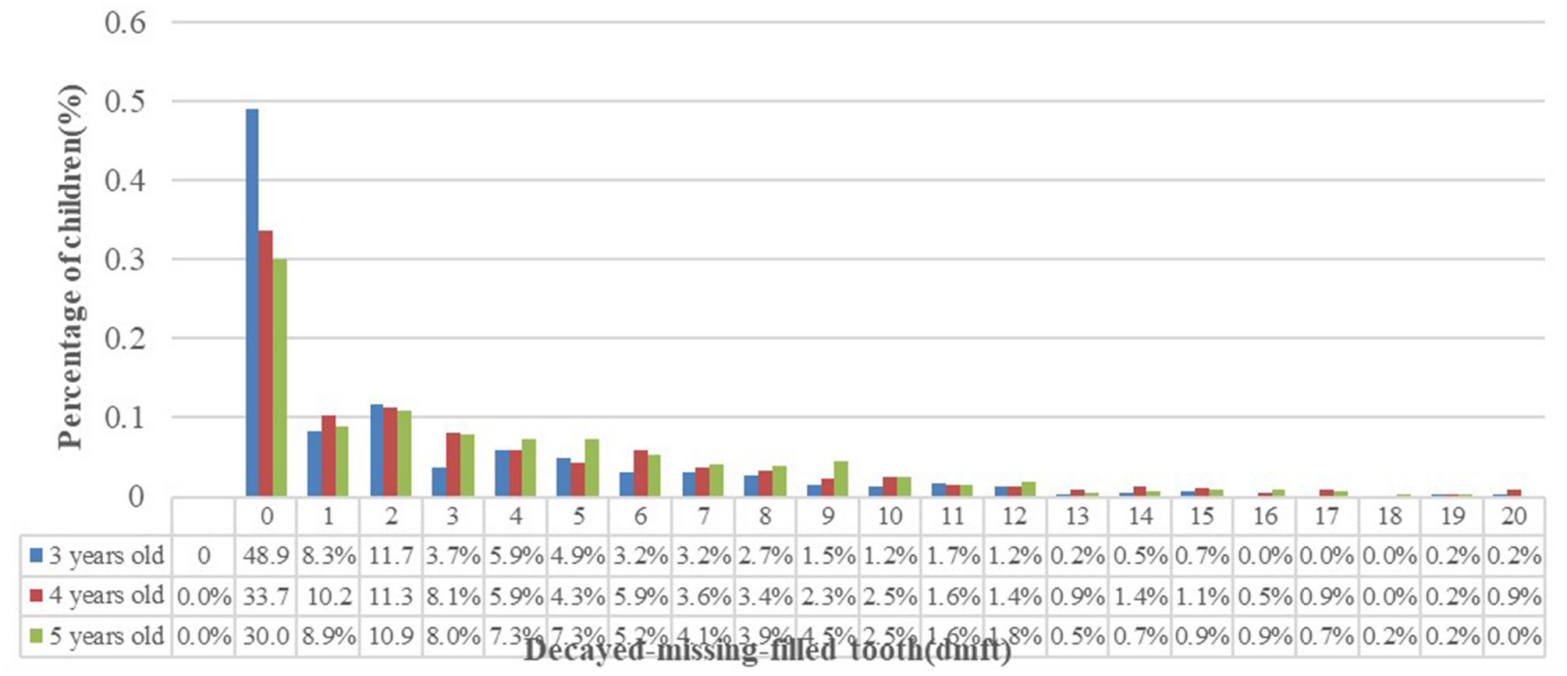
Distribution of the mean decayed-missing-filled teeth (dmft) frequency in deciduous teeth in children aged 3–5 years in Guizhou Province.

The frequency distribution of average caries in deciduous teeth is shown in Figure 1, with the majority of caries in two teeth, accounting for 11.7, 11.3, and 10.9% in the groups of children aged 3, 4, and 5 years, respectively, in all the examined children. The distribution of the number of participants gradually decreases with the increase.

**Figure 2:**
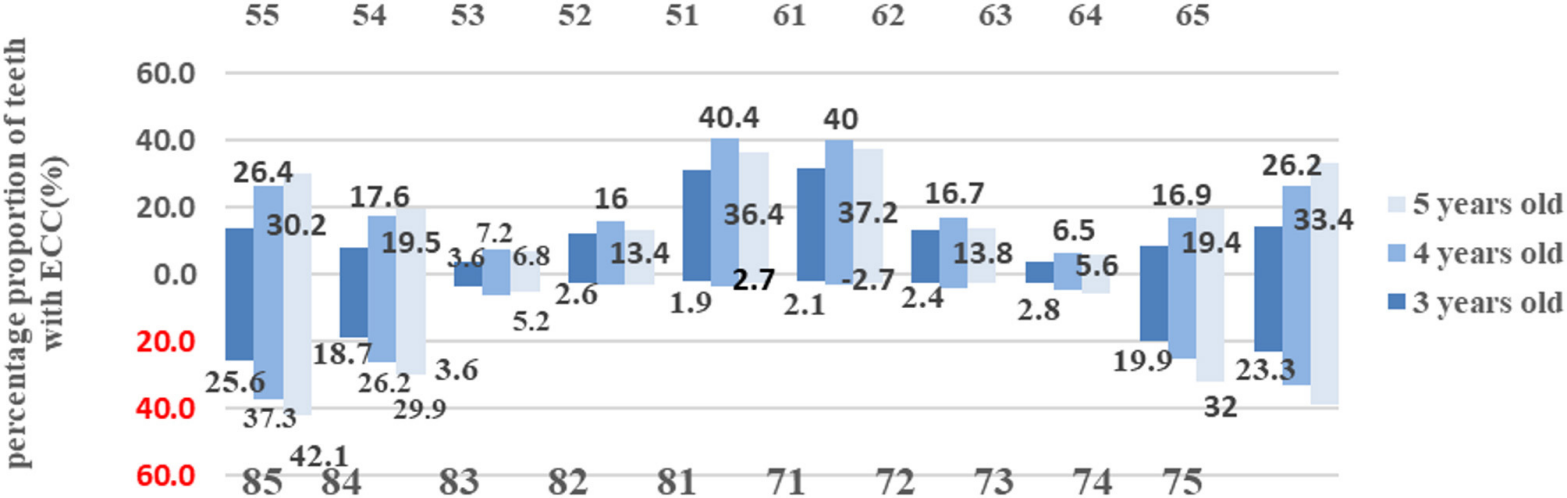
The distribution of primary caries in each tooth position in children aged 3–5 years in Guizhou Province.

The distribution of ECC (see Figure 2) showed that the most frequent caries sites were in the descending order that are as follows: the maxillary deciduous incisors, mandibular deciduous molars, and the maxillary deciduous molars. The children with age groups 3 and 4 years had the two maxillary deciduous incisors as the most frequently carious teeth. Moreover, the caries frequency in the deciduous molars increased gradually with age advancement, where the group of children aged 5 years had the highest caries prevalence in mandibular deciduous molars.

### Analysis of Caries-Related Factors

In the results of bivariate analysis related to caries (see Table 2), there were statistically significant differences (*p* < 0.05) between the brushing frequency per day, if the child had a toothache history in the past 12 months, if the parents took the child to the dentist, the parent evaluation of the oral condition of the child, the education of parents, and the advancement in age, and if the child had caries.

**Table 2:** A single-factor analysis of factors associated with early childhood caries (ECC) in children aged 3–5 years in Guizhou Province.

**Investigation factors**		**Number of people inspected (*n*, %)**	**Number of people suffering from the disease**	**Prevalence (%)**	**χ^2^ value**	***P*** **-value**
How the child is fed 6 months postnatally	Breastfed	827 (64.1)	535	64.7	4.11	0.128
	Artificially fed	319 (24.7)	186	58.3		
	Mixed feeding	145 (11.2)	93	64.1		
Desserts and sugar consumption	Daily ≥ 1 time	321 (24.9)	209	65.1	0.776	0.378
	Daily <1 time	970 (75.1)	605	62.4		
Sugary drink:s consumption	Daily≥1 time	176(13.6)	119	67.6	1.82	0.177
	Daily <1 time	1,115 (86.4)	695	62.3		
Consumption of dairy products: yogurt, milk powder, and milk tea	Daily ≥ 1 time	285 (22.1)	181	63.5	0.033	0.856
	Daily <1 time	1,006 (77.9)	633	62.9		
Consumption of sweets or drinks before bed	Present	93 (7.3)	56	59.6	0.526	0.468
	Absent	1,197 (92.7)	758	63.3		
Age of brushing onset	≥2 years old	832 (64.4)	536	64.6	3.886	0.143
	<2 years old	41 (3.2)	29	70.7		
	Unknown	418 (32.4)	249	59.6		
Brushing frequency per day	≥2 times	233 (18.0)	166	71.2	8.192	0.004
	<2 times	1,058 (82.0)	648	61.2		
Fluoridated toothpaste	Present	58 (4.5)	37	63.8	0.434	0.805
	Absent	163 (12.6)	99	60.7		
	Unknown	1,070 (82.9)	678	63.4		
History of toothache in the last 12 months	Absent	1,053 (81.6)	618	58.7	49.223	0.000
	Occasionally	171 (13.2)	143	83.6		
	Often	28 (2.2)	25	89.3		
	Unknown	39 (3.0)	28	71.8		
Parents taking their children to the dentist	Present	163 (12.6)	135	82.8	31.299	0.000
	Absent	1,128 (87.4)	679	60.2		
Parents' assessment of their child's oral condition	Good	832 (64.5)	466	56.0	75.528	0.000
	Average	328 (25.4)	225	68.6		
	Poor	131 (10.1)	123	93.9		
Parents' oral awareness attitude	0–4 points	129 (10.0)	79	61.2	0.237	0.888
	5–7 points	182 (14.1)	114	62.6		
	8–12 points	980 (75.9)	621	63.4		
The level of parental oral education	0–2 points	213 (16.5)	127	59.6	1.673	0.433
	3–5 points	735 (56.9)	473	64.4		
	6–8 points	343 (26.6)	214	62.4		
Household income	≥50,000 yuan	861 (66.7)	552	64.1	1.246	0.264
	>50,000 yuan	430 (33.3)	262	60.9		
Parental education	≤ 9 years	888 (68.8)	570	64.2	8.281	0.016
	10–15 years	292 (22.6)	188	64.4		
	≥16 years	111 (8.6)	56	50.5		

The factors that were statistically significant in the aforementioned analysis included in the LR model and analyzed by the Forward LR method and the results suggested that the caries prevalence increased with the advancement of ages of children; with the risk factors being the brushing frequency of <2 times a day and parents not taking their children to visit a dentist. Moreover, the worse the parents evaluation of the oral cavity of their children, the higher the caries prevalence. Likewise, the higher the education of parents, the lower the deciduous teeth caries prevalence of children. The results are shown in Table 3.

**Table 3:** Logistics regression analysis of 1,291 children aged 3–5 years in Guizhou Province.

**Variable**		**β-value**	**Wald chi-squared**	***P*** **-value**	**OR**	**95% CI**
Age groups	3-year-old group					
	4-year-old group	0.511	7.437	0.006	1.666	1.154 ~ 2.405
	5-year-old group	0.714	14.622	0.000	2.043	1.417 ~ 2.947
Daily brushing frequency	≥2 times					
	<2 times	0.446	6.485	0.011	1.563	1.108 ~ 2.204
Parents rate their children's oral condition	Good					
	Average	0.522	8.816	0.003	1.686	1.194 ~ 2.380
	Poor	2.880	22.962	0.000	17.806	5.483 ~ 57.820
Parent's education	≤ 9 years					
	12–15 years	−0.031	0.030	0.863	0.969	0.681 ~ 1.380
	≥16 years	−0.662	6.800	0.009	0.516	0.314 ~ 0.848
Parents taking their children to the dentist	Present					
	Unknown	0.550	4.249	0.039	1.733	1.027 ~ 2.923

## Discussion

The results of this study showed that the caries prevalence and the mean dmft of ECC in children aged 3–5 years in Guizhou Province (63.1%, 3.32) were comparable to the national average (62.5%, 3.35). The caries filling rate was only 0.5% with most caries not effectively treated. The difference between the urban and rural areas was not found to be significant, which may be related to the small economic disparity between the urban and rural areas based on administrative divisions randomly selected in Guizhou, where the overall economy is not developed (11). Compared with the prevalence rate of 50.3% (dmft = 1.92) among 5-year-old children in the Third Oral Epidemiological Survey in 2005, both caries prevalence and severity are on the rise, and this phenomenon also appears in the eastern regions of China such as Jiangsu Province and Guangdong Province (12), central regions such as Henan Province, and the western regions such as Sichuan Province (13). However, the trend of change of caries in deciduous teeth during the decade of 2005–2015 in Beijing faced a rapid increase where the rate of caries was curbed after intervention (14). Moreover, the caries prevalence of 5-year-old children in Hong Kong showed a trend of change from the peak value of 63–51% during the 14 years of 1993–2017, and then a small increase to 55% (15). The experience of oral healthcare service programs for preschool children in Beijing and Hong Kong suggests that ECC can be reduced by effective caries prevention measures, but it is a slow and long-term process (15). The caries situation of the younger children in Guizhou Province has been more serious, and the prevention and treatment of caries in deciduous teeth should be granted attention from early childhood.

The results of caries frequency and caries location distribution showed that the majority entails three caries per child, and the affected teeth are mostly distributed symmetrically, with caries in bilateral molars increasing with age. Moreover, several reports concluded that the overall high caries prevalence prevailed in the upper anterior teeth and posterior teeth bilaterally (12, 16–20), with the main reasons being as follows: caries in anterior deciduous teeth may be related to their early eruption in the oral cavity, poor feeding habits, and failure to perform proper oral hygiene measures on time (21–23); whereas caries in molar teeth may be related to the deep fissures and fossae on the occlusal surfaces that are difficult to clean, resulting in lengthy plaque retention (24–26). Therefore, teeth with higher caries prevalence should be the key focus for caries prevention. According to research on caries prevention methods (27–34), the incidence of ECC can be effectively reduced through fluoride application intervention two times yearly and timely fissure sealing after the eruption of molars to protect teeth at a high caries risk in both the anterior and posterior regions.

In this study, results suggested that the ratio of children consuming sweets, sugary drinks, and sweet milk/yogurt once or more per day, in addition to regularly eating desserts or drinking sugary drinks before bedtime is lower than the national level. There was no statistical correlation between the habit of eating sweets or drinking sugary drinks at least once a day and the habit of eating sweets and drinking sugary drinks before bedtime, which we speculate may be related to the diet structure in Guizhou Province where the preference of population is more inclined to spicy and sour foods because Guizhou Province is a non-sucrose-producing area and the per capita sucrose consumption thereby measures at an overall lower level than China (35).

Some scholars believe that brushing <2 times a day may lead to caries (36). However, it was found in the results of this study that the proportion of children with caries who brushed teeth two times or more a day was higher than those who brushed teeth <2 times a day, indicating that perhaps a higher brushing frequency per day does not equate to good oral hygiene, but perhaps mastering a correct brushing technique could, in theory, be the variable relevant to effectively improving oral hygiene of children (37, 38). Simultaneously, we speculate that an increase in the brushing frequency may be a behavioral change resulting after caries since it has been reported that children with caries display a significant improvement in their brushing habits after having received dental treatment (39, 40).

The education of parents influences the prevalence of ECC of children (41–44), where the results of our study also confirm that the higher the level of parental education was, the lower the prevalence of early childhood caries. Moreover, it has been suggested that the lower levels of parental education can be associated with a decreased financial ability which contributes to compromised overall access to dental resources, namely, decreased opportunities for dental checkups and dental visits (45). It has also been presented that oral health education is strongly related to socioeconomic status, which plays a key role in the prevention of ECC (46). It is, therefore the case that parents with higher levels of education are more likely to have oral health education and are thereby more capable of control and prevent ECC through enhanced child supervision and guidance that include improved oral health and dietary habits. Therefore, the parent evaluation of the oral condition of their children and whether or not they take their children to the dentist are directly related to ECC, concluding that besides dentists, parental education and their mastery of oral healthcare methods (especially correct brushing methods) play a crucial role in controlling ECC.

In addition to behavior and perception, the results of our survey were consistent with the national survey results by displaying that the majority of parents (75.9%) portray positive attitudes toward oral health. However, in our survey, the positive attitude of parents did not lead to a corresponding decrease in the ECC prevalence of children, and there was no correlation between the parental oral education level and the caries situation of children. Although the gross national product of Guizhou Province has grown rapidly in the recent years and even though this rapid economic growth has changed lifestyles and habits of the people, the positive attitude of parents did not lead to changes in the oral health habits, indicating that the transition from the knowledge and positive attitudes to implementation is a lengthy process, thereby delaying the display of positive behavioral changes in both the parents and their children in Guizhou Province.

This survey is a cross-sectional sample survey, and the questionnaire is thereby subject to few limitations. However, it can still roughly explain the overall oral health situation, namely, oral hygiene and dietary habits, awareness of oral healthcare of the parents, among children aged 3–5 years in Guizhou Province in a specific period.

The prevalence of ECC in Guizhou Province is not optimistic that should be addressed by effective measures to prevent and control.

Effective measures need to be taken to educate children to master oral healthcare methods (such as correct brushing methods). Measures are also needed to enhance parental guidance of young children (especially those with a lower education level) and regularly monitor the oral health of young children to achieve early caries detection and thereby early treatment. Moreover, it is important to actively adopt caries prevention measures through topical fluoride applications and fissure sealants to effectively control the occurrence of ECC.

## Conclusions

This study finds a high ECC prevalence among children aged 3–5 years living in Guizhou Province, China. Although the prevalence of ECC in Guizhou Province matched other regions in China in 2005, it is below the WHO target of 50% caries-free by the year 2000 for 5-year-old children and this despite the undoubted increase in the overall wealth in the past 10 years of the Province. Age, brushing frequency per day, history of toothache in the last 12 months, parent assessment of the oral condition of their child, and positive oral health attitude of parents were the significant factors for the occurrence of ECC in 3- to 5-year-old preschool children in Guizhou Province, China. As children often follow the oral health behavior of their parents, interventions should be designed to educate families and change their attitudes toward oral healthcare.

## Data Availability Statement

The original contributions presented in the study are included in the article/supplementary material, further inquiries can be directed to the corresponding author/s.

## Author Contributions

MG and ON conceived the study, supervised the experiments, and drafted the manuscript. J-jW and J-lS evaluated data and prepared the manuscript. NL analyzed the data and revised the manuscript. T-mD analyzed the data and performed the data collection. L-mC conceived the study, designed the data evaluation, and prepared the manuscript. All authors contributed to the article and approved the submitted version.

## Funding

This work was part of the Public Science and Technology Research Funds Project (2015)—the Fourth National Oral Health Survey (201502002), and supported by the Program of Oral Health Epidemiological Survey in Guizhou Province (gzwjkj2019-1-173).

## Conflict of Interest

The authors declare that the research was conducted in the absence of any commercial or financial relationships that could be construed as a potential conflict of interest. The handling editor declared a shared affiliation, though no other collaboration, with one of the authors ON at the time of the review.

## Publisher's Note

All claims expressed in this article are solely those of the authors and do not necessarily represent those of their affiliated organizations, or those of the publisher, the editors and the reviewers. Any product that may be evaluated in this article, or claim that may be made by its manufacturer, is not guaranteed or endorsed by the publisher.
